# An asymptomatic giant extra-renal retroperitoneal angiomyolipoma: Case report

**DOI:** 10.1097/MD.0000000000031869

**Published:** 2022-12-09

**Authors:** Kusay Ayoub, Amine Rakab, Mosa Shibani, Haidara Bohsas, Hidar Alibrahim, Sarya Swed, Mohammed Amir Rais, Bisher Sawaf, Nihad Mahli

**Affiliations:** a Department of Surgery, Aleppo University Hospital, Aleppo, Syria; b Medicine, Weill Cornell Medical College, Qatar; c Faculty of Medicine, Syrian Private University, Damascus, Syria; d Faculty of Medicine, Aleppo University, Aleppo, Syria; e Faculty of Medicine of Algiers, University Algiers; f Internal Medicine Department, Hamad Medical Corporation, Doha, Qatar; g Department of surgery, Aleppo University Hospital, Aleppo, Syria.

**Keywords:** case report, giant extra-renal tumor, retroperitoneal angiomyolipoma

## Abstract

Angiomyolipoma is a benign mesenchymal tumor that consists of adipose tissue, muscle cells and blood vessel. Renal angiomyolipomas represent almost one percent of all renal tumors.

**Patient concerns:**

We reported a case of a 42-year-old woman complaining of mild abdominal pain with no other symptoms and no remarkable medical history.

**Diagnosis:**

Clinical examination was inconclusive and revealed a large, smooth, non-tender, and immovable mass in the right abdomen. Ultrasound examination confirmed the existence of a large, homogeneous, hyperechoic tissue mass. Abdominal multi-slice computed tomography (CT) scans also confirmed the presence of a well-rounded mass in the right abdomen. The histopathology tests confirmed the diagnosis of a large retroperitoneal mass.

**Interventions:**

The patient underwent a traditional laparotomy without complications to remove the tumor.

**Outcomes:**

The open surgery was the best option, and the patient’s condition improved due to the following-up.

**Lessons:**

Retroperitoneal extra-renal angiomyolipomas are extremely rare, and in this case, we document a case of retroperitoneal angiomyolipoma manifested with only mild abdominal pain in Syrian women.

## 1. Introduction

Angiomyolipoma (AML) is a benign renal neoplasm composed of adipose tissue, vascular, and smooth muscle tissue, which manifest in two types, the isolated AML and tuberous sclerosis-associated AML. The lesions are often bilateral and multiple, with nearly equal distributions for tuberous sclerosis between females and males. Furthermore, this lesion could occur in other organs such as the uterus, liver, abdominal wall, and fallopian tube.^[1]^ The computed tomography (CT) scan and magnetic resonance imaging (MRI) are the right choices for diagnosing this lesion.^[2]^ However, these tumors are surgically treated with partial nephrectomy or vascular embolization, and there are several research investigations on more conservative therapies with a greater success rate.^[3,4]^

Here, we report a rare case of a 42-year-old woman with asymptomatic giant extra-renal retroperitoneal angiomyolipoma.

## 2. Case description

**Figure 1.**
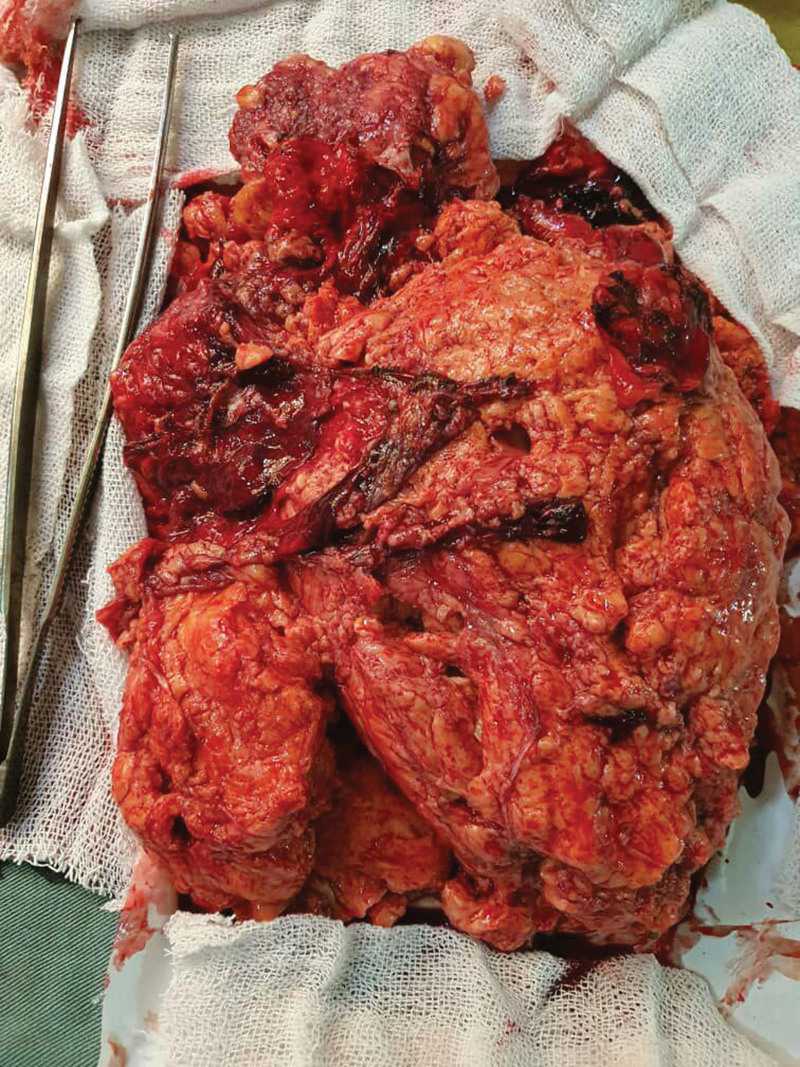
Gross appearance of the tumor after removing it and we can see the large size of this tumor and the gross adipose tissue and blood clots due to extensive tumor perfusion.

**Figure 2.**
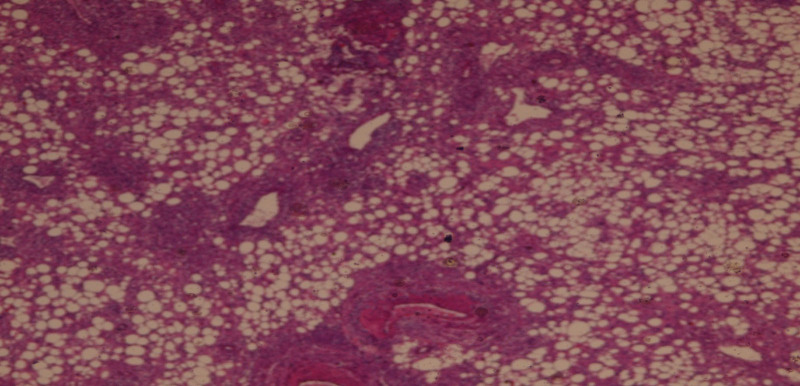
The histological appearance of the tumor after a guided needle biopsy showed mesenchymal proliferation made up of a mixture of smooth muscle fibers, mature fat tissue lobules, thick-walled blood vessels.

**Figure 3.**
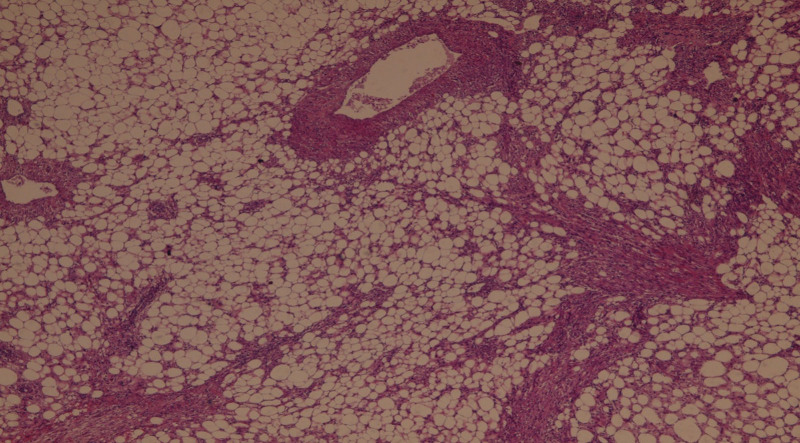
Pathological examination shows the combination of the three types of tissues, including muscle fibers, mature fat tissue lobules, and thick-walled blood vessels.

A 42-year-old Syrian woman came to Aleppo University Hospital complaining of mild abdominal pain with no other symptoms. Her medical history revealed a past thyroidectomy and a subsequent thyroxin replacement therapy. The Clinical examination showed a large, smooth, non-tender, and immovable mass in the right abdomen. Thus an abdominal ultrasound revealed a large, homogeneous, hyperechoic tissue mass composed of mainly adipose tissue. The mass was occupying the right hypochondrium and displacing the right kidney downward and medially. Abdominal multi-slice computed tomography (CT) scan showed a well-rounded mass, most of which is adipose tissue, with dimensions of (17 cm × 11 cm) at the expense of the superior lateral pole of the right kidney pushing it down and medially. Complete blood tests were within normal limits, and there were no tumor markers tests due to insufficient financial capabilities. A guided needle biopsy was performed, and the specimen was sent to the pathology evaluation. The pathology report revealed mesenchymal proliferation made up of a mixture of smooth muscle fibers, mature fat tissue lobules, and thick-walled blood vessels; thus, we suspected the diagnosis of retroperitoneal angiomyolipoma. The presence of the liposarcoma was not suspected because the patient’s general condition was good, and the abdominal computed tomography did not show any enlargement of the lymph nodes. After that, the patient underwent a traditional laparotomy without complications to remove the tumor (Fig. 1), and the tumor was sent to the pathology lab, and the immunohistochemistry (IHC) tests were performed, confirming the angiomyolipoma diagnosis. (Figs. 2 and 3) After that, the patient was followed up, and her general status improved after a week.

## 3. Discussion

The retroperitoneal space is considered one of the rarest places where extra-renal AML can appear.^[5]^ The tumor manifestations in this place vary according to its size, as it can give compressive symptoms such as obstruction of the ureter and hydronephrosis if it reaches a large size, in addition to digestive symptoms such as nausea, vomiting, constipation, and abdominal pain. AML has many differential diagnoses, the most important of which are liposarcomas, lipomas, leiomyosarcomas, leiomyomas, lymphomas, adenocarcinoma metastasis, and germ cell tumors. Therefore, multiple diagnostic tools become useful when diagnosing AML. The first imaging technique is ultrasound, which is sensitive specifically to adipose tissue within the tumor. However, Computed Tomography is more detailed, accurate, and rapid in the investigation. Other methods, including magnetic resonance imaging, are considered less harmful and safer for diagnosis, but they cannot be done quickly as CT. The final and definitive diagnosis is made by examining the biopsy that shows microscopic features of the tumor.^[6]^ In the medical literature, there are some cases similar to ours. Wroclawski et al documented a case of a 51-year-old patient who complained of right lumbar pain for one day. After conducting the appropriate tests, the patient underwent transperitoneal laparoscopy to remove the mass, and the diagnosis was a mesenchymal lesion compatible with AML.^[6]^ The case of Tseng et al documented the case of a Chinese woman who presented to the hospital with a complaint of increased waist circumference, and the clinical examination showed the presence of a painless mass with regular edges. After the patient underwent CT and ultrasound, AML was established as the initial diagnosis and confirmed by histopathology after surgical tumor removal.^[7]^

## 4. Conclusion

AMLs can sometimes be asymptomatic or present with mild or nonspecific symptoms, and therefore AMLs must be kept in mind when making a differential diagnosis of a kidney mass or retroperitoneal mass. The important role of ultrasound and CT in the early detection and investigation of these tumors should not be neglected.

## Acknowledgements

We would like to say thanks for Mohammad Ibn Abdullah regarding for his efforts in learning us.

## Author contributions

Kusay Ayoub, Mosa Shibani, Haidara Bohsas, Hidar Alibrahim, Sarya Swed, Hazem S. Ghaith, Karam R. Motawea, Lina Taha Khairy, Agyad Bakkour, Nihad Mahli: have contributed in writing and reviewing the manuscript. All authors reviewed and approved the manuscript for final publication.

**Writing – original draft:** Kusay Ayoub, Mosa Shibani, Haidara Bohsas, Hidar Alibrahim, Sarya Swed, Mohammed Amir Rais, Bisher Sawaf.

**Writing – review & editing:** Amine Rakab, Nihad Mahli.
